# Dr. August L. Gilbert

**Published:** 1906-03

**Authors:** 


Dr. August L. Gilbert, of North Cohocton, N. Y., a graduate of the University of Buffalo, 1884, died at his home, February 22, 1906, of pneumonia, aged 81 years.

